# Mitigating soil salinity–alkalinity and reshaping bacterial community to improve soil organic carbon sequestration in the Hetao Irrigation District: a combined approach of organic ameliorant and microbial agents

**DOI:** 10.3389/fpls.2026.1754594

**Published:** 2026-02-04

**Authors:** Ru Yu, Xia Zhang, Jie Zhou, Weini Wang, Jiashen Song, Fangdi Chang, Jing Wang, Haoruo Li, Xiaobin Li, Haigang Li, Hongyuan Zhang

**Affiliations:** 1 College of Resources and Environment sciences, Inner Mongolia Agricultural University, Inner Mongolia Key Laboratory of Soil Quality and Nutrient Resources, Hohhot, China; 2 State Key Laboratory of Efficient Utilization of Arable Land in China (the Institute of Agricultural Resources and Regional Planning), Chinese Academy of Agricultural Sciences, Beijing, China; 3 College of Agriculture, Nanjing Agricultural University, Nanjing, China; 4 Ordos Agriculture and Animal Husbandry Ecology and Resource Protection Center, Ordos, China; 5 National Center of Technology Innovation for Comprehensive Utilization of Saline-Alkali Land, Dongying, China

**Keywords:** carbon sequestration, microbial community, organic ameliorants, saline-alkali soil, soil organic carbon fractions

## Abstract

Microbial agents promote the decomposition and the transformation of organic materials, enhancing the utilization of organic ameliorants in saline–alkali soil. However, the effects of the combination of organic ameliorants and microbial agents on the soil organic carbon (SOC) pool and the underlying regulatory mechanisms are poorly understood. Consequently, a 2-year field experiment was conducted in 2022–2023 to investigate changes in the SOC fractions and pools, as well as the regulatory pathways under different organic ameliorants: no organic ameliorants (CK), sole organic ameliorants (O), and organic ameliorants combined with microbial agents (O+M). O+M was superior to O treatment in improving the soil saline–alkali and nutrient conditions, alleviating the microbial carbon (C) and phosphorus (P) limitation and increasing the bacterial diversity and richness in saline–alkali soil. Community analysis revealed that O+M preferentially enriched the K-strategists (Actinobacteriota, +16%; Acidobacteriota, +6%), increasing the bacterial K/r ratio by 9% compared with O treatment. Partial least squares path modeling (PLS-PM) identified distinct SOC accrual pathways: the O and O+M treatments directly enhanced SOC, the O treatment increased the particulate organic carbon (POC) via bacterial diversity-mediated nutrient cycling, whereas the O+M treatment promoted POC through K/r ratio shifts and the mineral-associated organic carbon (MAOC) via soil content and pH modulation. Ultimately, O+M achieved the highest SOC (8.26 g kg^−1^, +34% *vs*. CK), with MAOC contributing 32% of the total increment, 61% higher than that of O treatment. This mechanistic study elucidates how microbial–metabolic coordination and abiotic stabilization jointly regulate the persistence of SOC in degraded soils, providing a synergistic bioremediation strategy for saline–alkali ecosystems.

## Graphical Abstract

**Figure:**
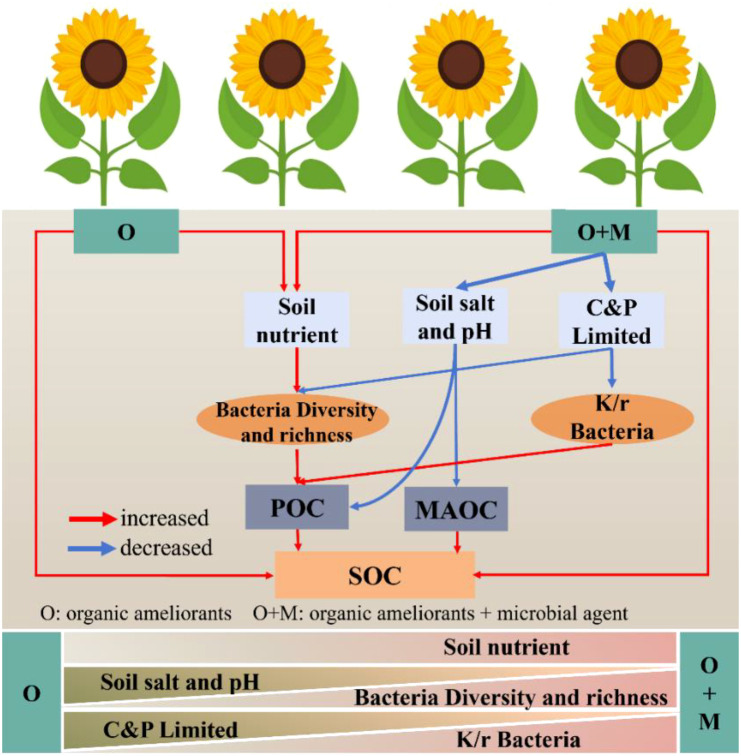

## Highlights

- Treatment with organic ameliorants combined with microbial agents (O+M) boosts the SOC by 10% compared with O treatment in saline–alkali soils.
- O+M induces a shift toward bacterial K-strategists driven by an improved nutrient availability and stoichiometric balance.
- The K-strategy dominance under O+M treatment promotes particulate organic carbon (POC) stability and mineral-associated organic carbon (MAOC) formation.
- MAOC contributes 32% of the SOC increment, with O+M treatment 61% higher than O treatment.

## 1 Introduction

Soil salinization affects over 8 × 10^8^ ha of global cropland (FAO, 2021), posing severe threats to agricultural, ecological, and environmental sustainability. Importantly, this leads to the substantial depletion of soil organic carbon (SOC), further constraining agricultural productivity (Lal, 2004; Setia and Marschner, 2013). A common remediation strategy involves the application of organic ameliorants (e.g., manure and humic acid), which can directly add exogenous carbon to soils (Liu et al., 2023). However, high salinity stress suppresses the microbial activity, limiting the efficient transformation of this exogenous carbon into stable SOC pools (Dong et al., 2022). The application of microbial agents offers a potential solution to mitigate this limitation. Such agents typically possess nitrogen-fixing and phosphorus/potassium-solubilizing capabilities, which can stimulate the microbial communities and alleviate saline–alkali stress (Bamdad et al., 2022; O’Callaghan et al., 2022; Yang et al., 2024). While the positive role of microbial agents in enhancing SOC sequestration has been demonstrated in non-saline soils (Srivastava and Singh, 2022), primarily by promoting organic matter decomposition and improving nutrient availability (Mason et al., 2023), their effects on carbon sequestration when combined with organic ameliorants in saline–alkali soils remain unclear.

The SOC pool is not homogeneous, but consists of distinct fractions formed and stabilized through different pathways (Angst et al., 2023). A widely adopted framework dichotomizes SOC into particulate organic carbon (POC) and mineral-associated organic carbon (MAOC) (Lavallee et al., 2020). POC consists mainly of plant-derived structural polymeric compounds and exhibits a fast turnover with less physical protection (Witzgall et al., 2021; Guo et al., 2022). MAOC primarily comprises microbial-derived compounds that stabilize through associations with soil minerals, thereby gaining resistance to decomposition (Cotrufo et al., 2019). In saline–alkali soils, high salinity hinders soil particle cementation, reducing the POC content (Hassani et al., 2024), while saline ions (e.g., Na^+^, HCO_3_^−^, and Cl^−^) occupy the binding sites on soil minerals and organic materials, impeding the formation of MAOC (Kleber et al., 2021; Chi et al., 2022). Microbial agents have been applied to soil: i) promoting organic matter decomposition and plant growth, which increases the production of organic acids, and ii) improving the soil nutrient availability and physical structure, thereby facilitating salt leaching and subsequently reducing the soil salinity barriers (Cui et al., 2019). Whether microbial agents promote the formation of POC and MAOC by alleviating soil saline–alkali stress under organic ameliorants and how the contributions of these fractions to SOC sequestration differ remain to be verified.

Soil microorganisms are the primary drivers of SOC cycling as their community composition and extracellular enzyme activity collectively determine SOC turnover (Liu et al., 2022). Combining organic ameliorants with microbial agents supplies substantial carbon and energy substrates for the soil microbiome, stimulating microbial growth and the secretion of extracellular enzymes (Bebber and Richards, 2022; Zhang et al., 2024). Furthermore, microbial agents also solubilize insoluble soil nutrients, thereby enhancing nutrient accessibility and indirectly promoting microbial proliferation and enzymatic activity (Mason et al., 2023). In addition, the application of microbial agents can selectively enrich keystone taxa, restructuring the soil microbiome through the modulation of community assembly processes (Li et al., 2024a; Bamdad et al., 2022). These shifts in community structure are critical because different microbial groups employ distinct resource acquisition strategies, which in turn influence the SOC sequestration pathways. For instance, copiotrophic r-strategists (e.g., Proteobacteria and Bacteroidetes) exhibit a low utilization efficiency of organic matter and prefer to use labile organic matter. Oligotrophic K-strategists (e.g., Actinobacteriota, Acidobacteriota, and Blastomonas) possess a high utilization efficiency of organic matter and mainly decompose recalcitrant organic matter (Fierer et al., 2007; Malik et al., 2020). Importantly, the soil microbiome composition mediates the formation of SOC fractions. Specifically, microorganisms increase the POC content by promoting the association of plant-derived macromolecules with soil particles through extracellular modification. Concurrently, through microbial turnover (e.g., via cellular secretions and necromass), microorganisms yield persistent microbial products that serve as key precursors for MAOC formation (Xiao et al., 2023). Therefore, clarifying the regulatory pathways of SOC sequestration necessitates identifying shifts in the soil microbial communities and its subsequent regulation of the POC and MAOC dynamics under synergistic organic–microbial amendments.

While the application of microbial agents has been widely studied as a promising practice for salt-affected soil amelioration, the majority of studies have focused on their effects on soil nutrient availability, crop growth, and stress resistance (Bamdad et al., 2022; O’Callaghan et al., 2022; Mason et al., 2023). However, the effects of the combination of microbial agents with organic ameliorants on the SOC pools and their underlying regulatory mechanisms in saline–alkali soils remain unclear. This knowledge gap limits our ability to enhance agronomic productivity and ensure the long-term sustainability of salt-affected soil amelioration. We therefore conducted a 2-year field trial (2022–2023) to: i) quantify changes in the SOC fractions (POC and MAOC) in saline–alkali soil under organic ameliorants combined with microbial agents and ii) determine the biotic and abiotic regulatory pathways that govern SOC formation under this combined treatment. We hypothesized that the application of microbial agents would increase the POC, MAOC, and SOC contents under organic ameliorants in saline–alkali soil, mediated through alleviating the salinity stress, enriching the nutrient supply, and optimizing the microbial community structure.

## 2 Materials and methods

### 2.1 Experimental site

A field experiment was conducted in 2022 at Beihaizi Research Station (40.48° N, 109.87° E) in Dalate Banner, Ordos City, Inner Mongolia. Characterized by a semi-arid continental monsoon climate, the site exhibits mean annual temperatures of 6.1–7.1°C, 150–170 frost-free days, and annual precipitation of 240–360 mm. The experimental plots established on anthropogenic-alluvial soils exhibited the following initial properties (0- to 20-cm depth): pH 8.5; salt content, 1.41 g kg^−1^; SOC, 6.93 g kg^−1^; total nitrogen (TN), 0.73 g kg^−1^; available nitrogen (AN), 0.06 g kg^−1^; available phosphorus (AP), 19 mg kg^−1^; and available potassium (AK), 131 mg kg^−1^.

### 2.2 Experimental design and material

With a randomized block design, the field experiment was designed with three fertilization treatments in a sunflower continuous cropping system: no organic ameliorants (CK), with organic ameliorants (O), and with organic ameliorants combined with microbial agents (O+M). Each treatment was replicated five times, and each plot measured 665 m^2^ (length = 95 m, width = 7 m). The organic material was a mixture of cow manure and humic acid, with application rates of 15 and 1.5 t ha^−1^, respectively. The application rate of microbial agents was 45 kg ha^−1^. The organic material and microbial agents were applied before sowing and then mixed with the soil (0- to 10-cm depth) by rotary tillage. Cow manure contained 163.1 g kg^−1^ organic carbon, 15.4 g kg^−1^ nitrogen (N), 3.3 g kg^−1^ phosphorus (P), and 22.5 g kg^−1^ potassium (K). Humic acid contains 345.5 g kg^−1^ organic carbon, 10.1 g kg^−1^ N, 3.2 g kg^−1^ P, and 1.2 g kg^−1^ K. The microbial agents used in this study were a commercially available compound microbial inoculant [microbial fertilizer registration permit no. (2014) 1405; Yuanhe Biotechnology (Dezhou) Co., Ltd., China], in compliance with the Chinese National Standard GB20287-2006. According to the manufacturer’s specifications, the microbial agents demonstrated a minimum viable bacterial concentration of 200 million per gram and consisted primarily of *Bacillus subtilis*, *Bacillus amyloliquefaciens*, and Actinomycetes.

Sunflowers (*Helianthus annuus* L.) were planted annually in May with wide–narrow rows. The wide and narrow row spacings were 1.0 and 0.4 m, respectively, achieving a planting density of 49,000 plants ha^−1^. Sunflowers were harvested in October each year. Base fertilization consisted of 375 kg ha^−1^ diammonium phosphate and 180 kg ha^−1^ potassium sulfate. An additional 450 kg ha^−1^ urea was top-dressed when the sunflower plants reached the big trumpet period. All treatments were mulched using agricultural plastic film. Other farming practices were consistent with the local practices in this experiment.

### 2.3 Sampling and measurement

Following the October 2023 sunflower harvest, composite 0- to 20-cm soil samples were collected from five random positions per plot. After removing mulch, stones, and plant debris, the samples were divided into three subsamples. One was air-dried for the analysis of soil salt, pH, nutrients, and the SOC fractions; another was refrigerated at 4°C for enzyme activity assays within 2 weeks; and the last subsample was stored at −80°C for determination of the soil microbial community composition. SOC was determined with the K_2_Cr_2_O_7_ colorimetric oxidation method (Bao, 2000). TN was measured using Kjeldahl digestion, while AP was quantified via the molybdenum antimony colorimetric method. AK was analyzed using a flame photometer, and AN was determined with the alkaline hydrolysis diffusion method. For the pH and electrical conductivity (EC) analysis, the soil was homogenized with deionized water (1:5) and measured using a PHS-3B pH meter and a DDS-307 conductivity meter, respectively. The soil salt content was equal to the EC multiplied by a coefficient of 3.0111 according to previous studies (Zhang et al., 2020). The POC and MAOC were determined according to Zhang et al. (2024).

Relative contribution reveals whether carbon sequestration is primarily driven by the relatively transient POC pool or the more stable MAOC pool. A higher relative contribution from MAOC indicates that a greater proportion of the newly sequestered carbon will be protected, exhibit greater stability, and persist in the soil over the long term. The relative contribution was calculated using Equations 1 and 2:

(1) $$Relative\;contribution\left(POC\right)=\frac{\Delta POC}{\Delta SOC}\times 100\%$$

(2) $$Relative\;contribution\left(MAOC\right)=\frac{\Delta MAOC}{\Delta SOC}\times 100\%$$

where ΔSOC, ΔPOC, and ΔMAOC represent the increase in each carbon pool for the O or the O+M treatment relative to CK. The relative contribution of each fraction was then calculated based on these changes.

#### 2.3.1 Soil enzyme activity analysis

The activities of the C-related enzymes [β-1,4-glucosidase (BG) and cellobiosidase (CBH)], the N-related enzymes [β-1,4-*N*-acetyl-glucosaminidase (NAG) and leucine aminopeptidase (LAP)], and the P-related enzyme [alkaline phosphatase (AP)] were determined using fluorescence microplate enzyme detection technology on a 96-well plate (Marx et al., 2001). Fluorescence was quantified at 365 and 450 nm excitation/emission wavelengths using a Multiscan Sky microplate reader (Multiscan Sky 1510, Thermo Scientific, Waltham, MA, USA).

The soil enzyme stoichiometric parameters were used to evaluate the microbial resource limitation based on Equations 3 and 4 (Song et al., 2023):

(3) $$Vector\;length=\sqrt{{\left[\frac{\ln\left(BG+CBH\right)}{\ln\left(NAG+LAP\right)}\right]}^{2}+{\left[\frac{ln\left(BG+CBH\right)}{lnALP}\right]}^{2}}$$

(4) $$Vector\;angle=Degrees\left\{ATAN2{\left[\frac{ln\left(BG+CBH\right)}{ln\left(NAG+LAP\right)}\right]}^{2},{\left[\frac{ln\left(BG+CBH\right)}{lnALP}\right]}^{2}\right\}$$

where the vector length represents the C limitation, with larger values indicating higher C limitations. The vector angle indicates N or P limitation, where angles greater than 45° denote P limitation and angles less than 45° denote N limitation.

#### 2.3.2 Soil microbial community structure analysis

The soil microbial community structure was analyzed using high-throughput sequencing technology (Biddle et al., 2008; Bellemain et al., 2010). Details are shown in the **Supplementary Material**. At the phylum level, for bacteria, Gemmatimonadota and Bacteroidota were categorized as r-strategists, while Acidobacteriota, Actinobacteriota, Chloroflexi, and Planctomycetota were categorized as K-strategists (Li et al., 2024d). For fungi, Ascomycota and Mortierellomycota were classified as r-strategists, while Basidiomycota was categorized as a K-strategist (Chang et al., 2025).

### 2.4 Statistical analyses

The effects of the fertilization treatments (i.e., CK, O, and O+M) on the soil pH, salt content, nutrients, SOC, POC, MAOC, enzyme activities, vector length, vector angle, and microbial community were analyzed using one-way analysis of variance (ANOVA) (Duncan’s multiple range test: *p <* 0.05). The data are presented as the mean ± standard error (*n* = 5). Pearson’s correlation analysis was used to assess the relationships among the soil carbon fractions, soil salt contents, nutrients, enzyme activities, and microbial community. Partial least squares path modeling (PLS-PM) quantified the contribution of the soil salt content, nutrients, microorganisms, and microbial resource limitation to the SOC pool under fertilization treatments using the R package “plspm.” Goodness of fit was used to estimate the model prediction performance. Figures were obtained using Origin 2021.

## 3 Results

### 3.1 Soil nutrient, salt, and pH

**Figure 1:**
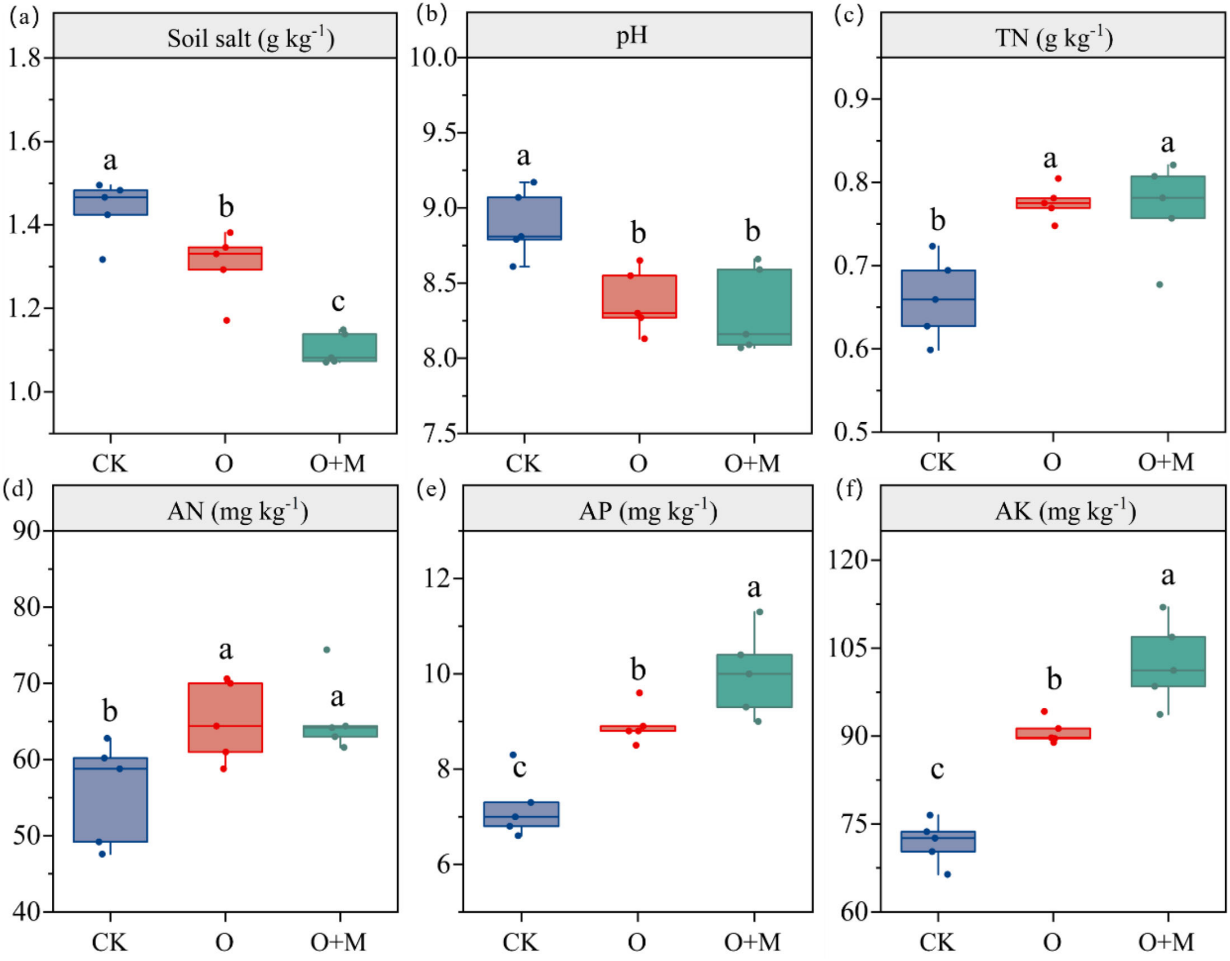
Differences in the soil salt **(A)**, pH **(B)**, total nitrogen **(C)**, available nitrogen **(D)**, available phosphorous **(E)**, and available potassium **(F)** under different treatments. *TN*, total nitrogen; *AN*, available nitrogen; *AP*, available phosphorous; *AK*, available potassium. *CK* denotes no organic ameliorants, *O* represents organic ameliorants, and *O+M* indicates organic ameliorants combined with microbial agents. *Different lowercase letters* indicate significant differences between treatments (*p* < 0.05).

Compared with CK, the O and O+M treatments increased TN by 17% and 16%, AN by 17% and 18%, AP by 24% and 39%, and AK by 26% and 43%, respectively (*p <* 0.05) (**Figure 1**). The O and O+M treatments also decreased the soil salt content by 9% and 23% and the pH by 5% and 6%, respectively (*p <* 0.05). Compared with the O treatment, O+M decreased the salt content by 15% and increased the AP and AK by 12% and 13%, respectively (*p <* 0.05).

### 3.2 POC, MAOC, and SOC contents

**Figure 2:**
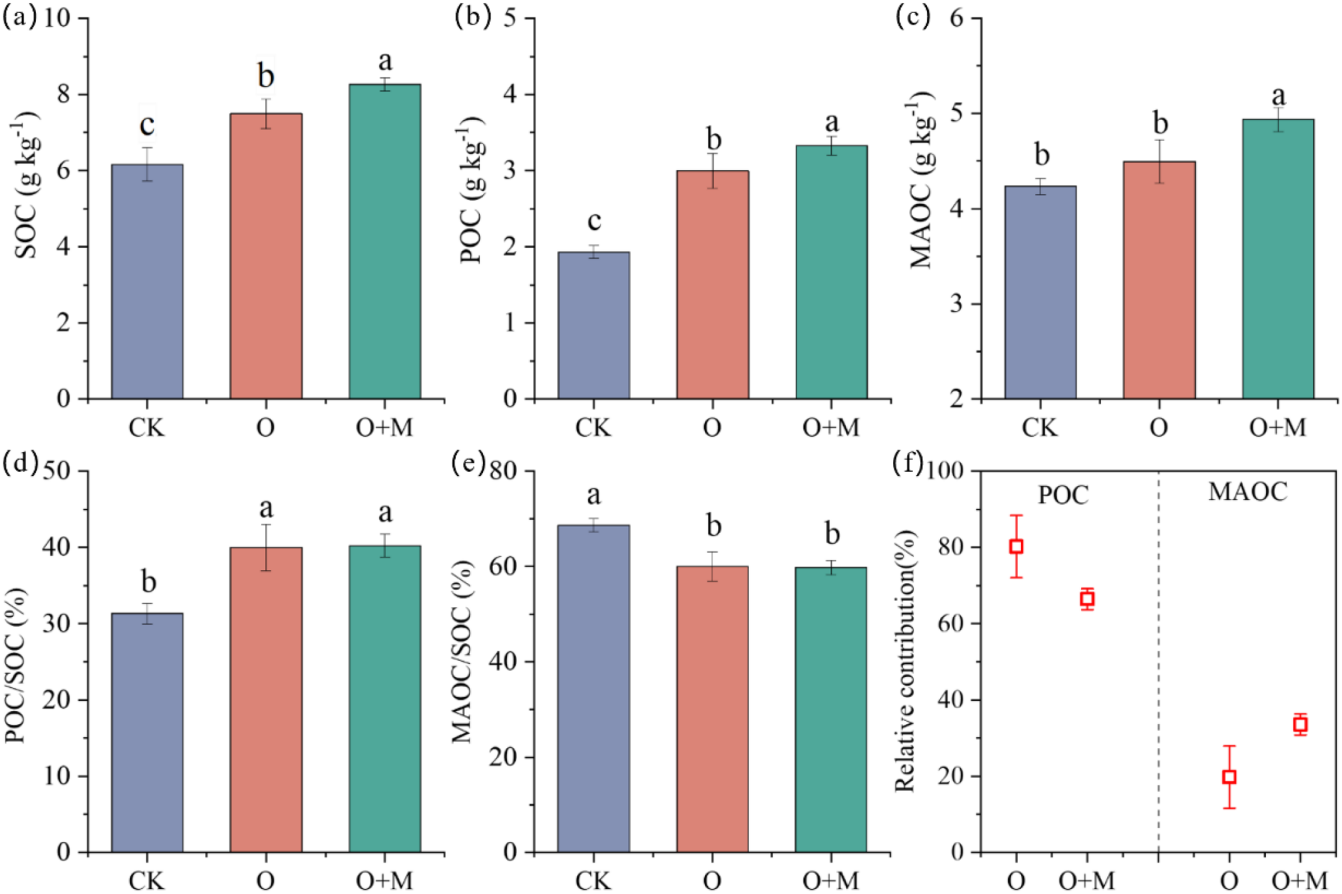
Differences in the SOC **(A)**, POC **(B)**, MAOC **(C)**, POC/SOC **(D)**, and MAOC/SOC **(E)** and the relative contribution of POC and MAOC to SOC **(F)** under different treatments. *SOC*, soil organic carbon; *POC*, particulate organic carbon; *MAOC*, mineral-associated organic carbon. *CK* denotes no organic ameliorants, *O* indicates organic ameliorants, and *O+M* denotes organic ameliorants combined with microbial agents. *Different lowercase letters* indicate significant differences between treatments (*p* < 0.05).

Compared with CK, the O and O+M treatments increased the SOC content by 22% and 34% and the POC content by 55% and 72%, respectively (*p <* 0.05) (**Figures 2A, B**). Moreover, the O+M treatment also increased the MAOC content by 17% relative to CK (**Figure 2C**). Compared with the O treatment, O+OM increased the SOC, POC, and MAOC by 10%, 11%, and 10%, respectively (*p <* 0.05) (**Figure 2**). POC and MAOC contributed 66%–80% and 20%–34% of the SOC increment under organic ameliorants, respectively (**Figures 2D, E**). O+M elevated the contribution of MAOC to SOC by 61% relative to the O treatment (*p <* 0.05) (**Figure 2F**).

### 3.3 Soil enzyme activity and stoichiometry

**Figure 3:**
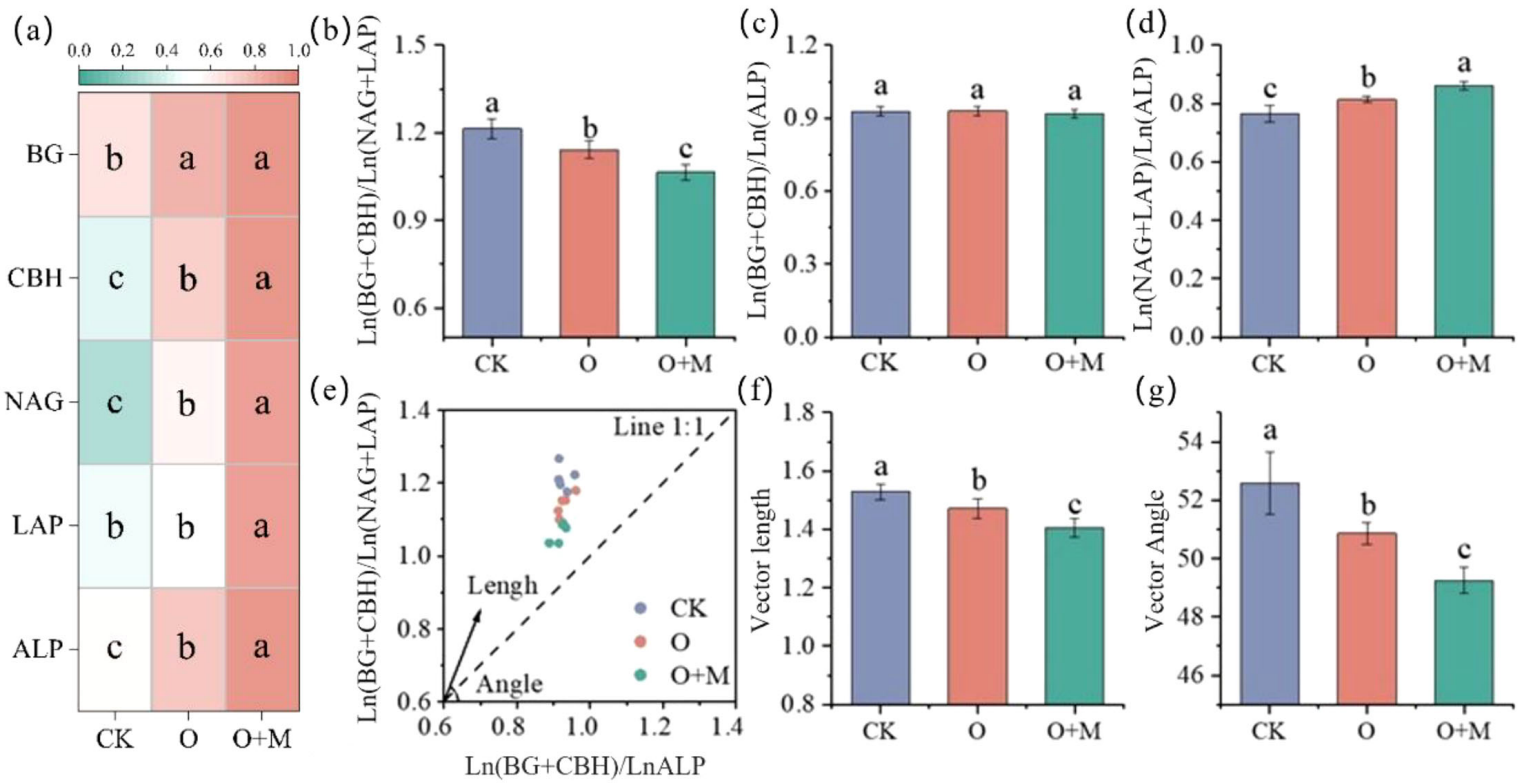
The *Z*-score normalized enzyme activities **(A)** and soil enzyme stoichiometry **(B–G)** under different treatments. *CK* indicates no organic ameliorants, *O* represents organic ameliorants, and *O+M* denotes organic ameliorants combined with microbial agents. *BG*, β-1,4-glucosidase; *CBH*, cellobiosidase; *NAG*, β-1,4-*N*-acetyl-glucosaminidase; *LAP*, leucine aminopeptidase; *ALP*, alkaline phosphatase. *Different lowercase letters* indicate significant differences between treatments (*p <* 0.05).

Compared with the CK treatment, O and O+M increased the BG, GBH, NAG, and AP activities by 26%–41%, 68%–111%, 104%–210%, and 33%–65%, respectively (*p <* 0.05) (**Figure 3A**). O+M increased the GBH, NAG, LAP, and ALP activities by 25%, 52%, 75%, and 24%, respectively, relative to O (*p <* 0.05) (**Figure 3A**). Compared with that of CK, the vector lengths of the O and O+M treatments decreased by 4% and 8%, respectively (*p <* 0.05) (**Figure 3F**), alleviating the soil microbial C limitation. The O+M treatment decreased by 5% relative to O (*p <* 0.05). The vector angles were >45°, indicating that the soil microbial P limitation was recorded in all treatments (*p <* 0.05) (**Figure 3G**).

### 3.4 Microbial community composition

**Figure 4:**
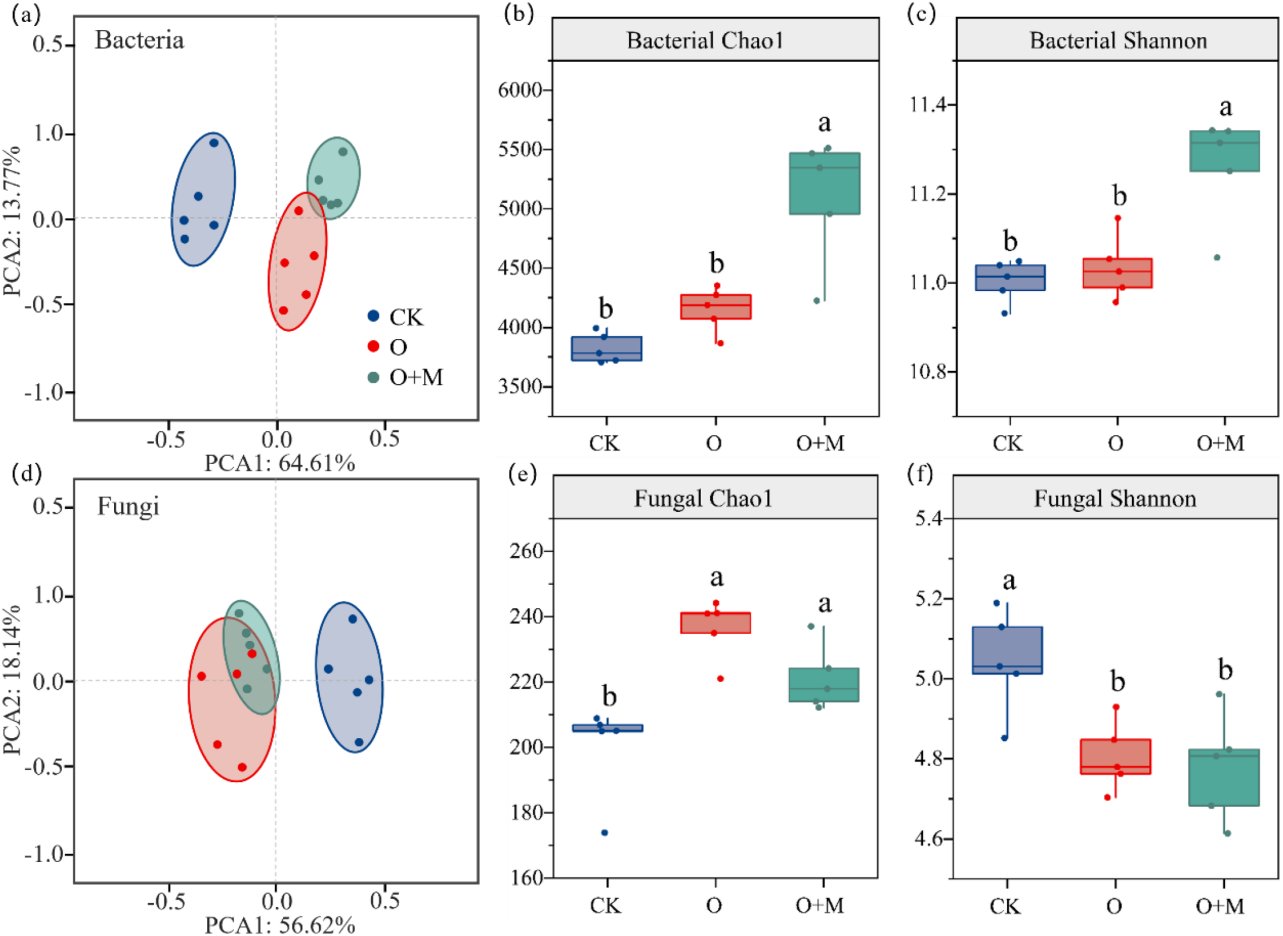
Principal component analysis (PCA) of the microbial community structure **(A, D)** and the Chao1 and Shannon indices of bacteria **(B, C)** and fungi **(E, F)** under different treatments. *CK* denotes no organic ameliorants, *O* indicates organic ameliorants, and *O+M* represents organic ameliorants combined with microbial agents. *Different lowercase letters* indicate significant differences between treatments (*p <* 0.05).

**Figure 5:**
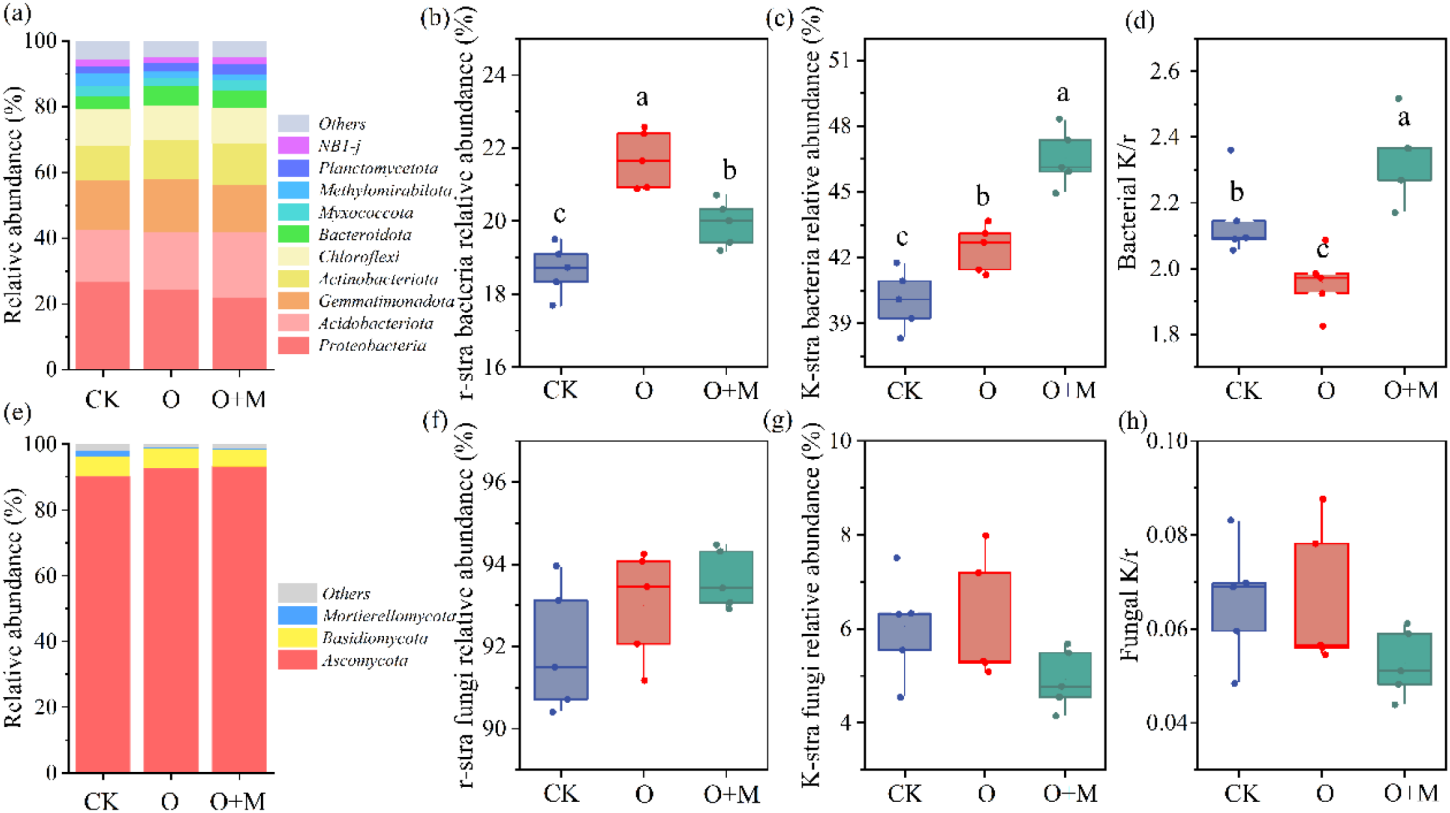
Relative abundance of bacteria **(A)** and fungi **(E)** at the phylum level and its implications for the r/K-strategy of bacteria **(B–D)** and fungi **(F–H)** under different treatments. *CK* denotes no organic ameliorants, *O* represents organic ameliorants, and *O+M* indicates organic ameliorants combined with microbial agents. *Different lowercase letters* indicate significant differences between treatments (*p <* 0.05).

Relative to CK and O, the O+M treatment increased the Chao1 of bacteria by 23% and 33% and the Shannon index of bacteria by 2% and 2%, respectively (*p* < 0.05) (**Figures 4B, C**). The O and O+M treatments increased the Chao1 of fungi by 18% and 11%, respectively (*p <* 0.05) (**Figure 4E**), both treatments significantly reduced Shannon of fungi (**Figure 4F**). The distribution of the soil bacterial and fungal communities varied with treatments, with those of the O and O+M treatments being quite distinct from those of CK (**Figures 4A, D**). In the bacterial group, Proteobacteria, Acidobacteriota, Gemmatimonadota, Actinobacteriota, and Chloroflexi were the dominant phyla, accounting for over 75% of the bacterial abundance (**Figure 5A**). Compared with CK, the O and O+M treatments increased the relative abundance of Acidobacteriota and Actinobacteriota by 10%–27% and 13%–19%, respectively, and decreased the relative abundance of Proteobacteria by 9%–18% (*p <* 0.05) (**Figure 5A**). The O+M treatment increased Actinobacteriota and Acidobacteriota by 6% and 16%, respectively, relative to O (*p <* 0.05) (**Figure 5A**). In the fungal group, Ascomycota, Basidiomycota, and Mortierellomycota were the dominant phyla, accounting for over 95% of the fungal abundance (**Figure 5E**). Compared with CK, the O and O+M treatments increased Ascomycota, but decreased Mortierellomycota (*p <* 0.05) (**Figure 5E**).

The soil microorganisms were divided into K-strategist and r-strategist microorganisms according to their nutrient utilization strategies (**Figure 5**). The relative abundance of r-strategist bacteria showed a trend of O > O+M > CK (**Figure 5B**). The relative abundance of K-strategist bacteria showed a trend of O+M > O > CK (**Figure 5C**). Compared with CK, the O treatment decreased the K/r strategy of bacteria by 9%, while the O+M treatment increased the K/r strategy of bacteria by 9% (*p <* 0.05) (**Figure 5D**). The O+M treatment increased the relative abundance of K-strategist bacteria and the K/r strategy of bacteria by 10% and 19%, respectively, relative to O (*p* < 0.05) (**Figures 5C, D**). The effects of treatment on K/r-strategist fungi were non-significant (**Figures 5F, G**).

### 3.5 Effects of environmental factors on the SOC pool

**Figure 6:**
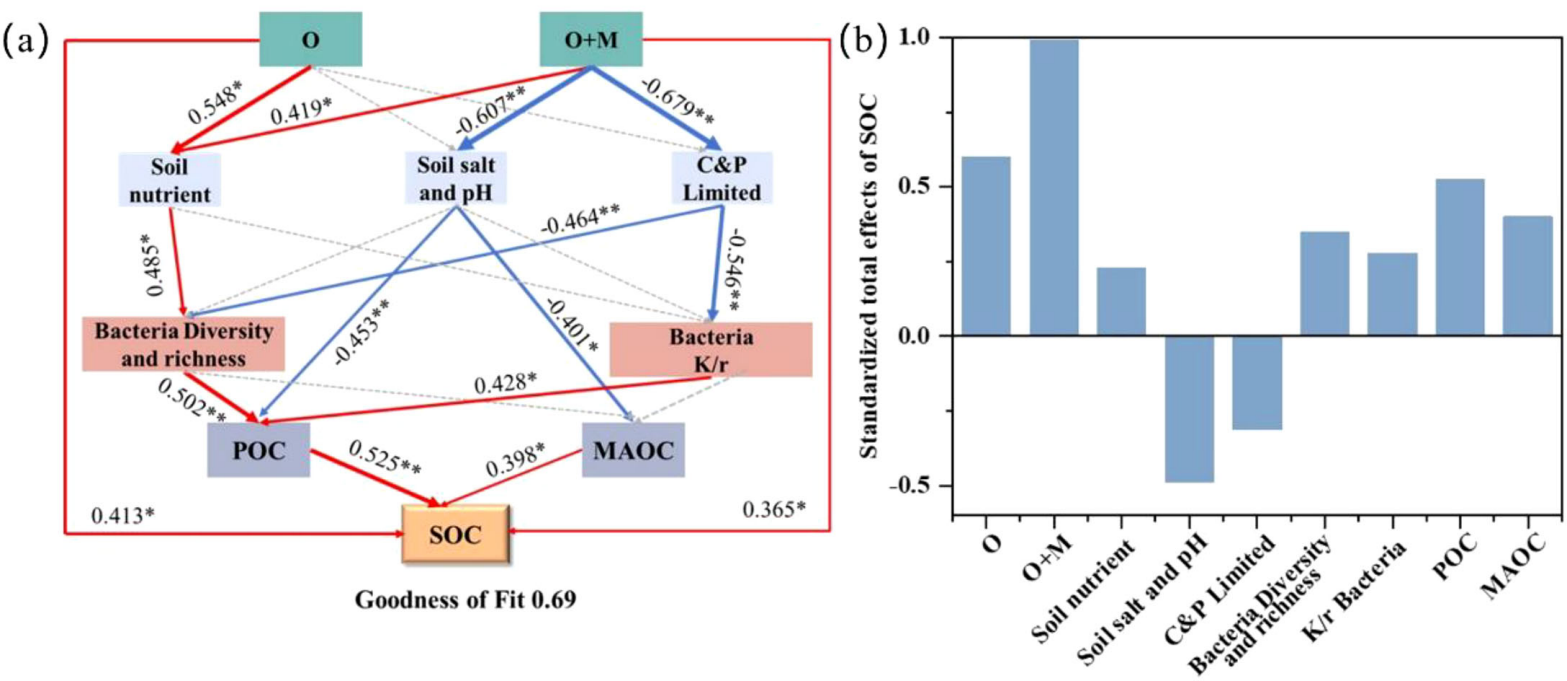
A partial least squares path model **(A)** and the standardized total effects **(B)** describing the effects of the key factors on the soil organic carbon (SOC) pool. Significance level of each predictor: **p < 0.01, *p < 0.05. *Red* and *blue arrows* represent significant positive and negative correlations, respectively (*p* < 0.05). The *width of arrows* denotes the correlation intensity. *Numbers next to the lines* are the correlation coefficients.

The soil nutrients, salt, pH, microbial C limitation, microbial P limitation, and bacterial group primarily regulated the SOC pool, which had significant correlations with SOC, POC, and MAOC (**Supplementary Figure S1**). To quantify the regulatory pathways governing the SOC pool responses to these factors, PLS-PM was conducted (**Figures 6A, B**). The results showed that both the O [standard path coefficient (ρ) = 0.413, *p <* 0.05] and O+M (ρ = 0.365, *p <* 0.05) treatments had significant direct effects on SOC. Furthermore, both treatments enhanced SOC indirectly via bacterial-mediated POC formation. Specifically, the O treatment (ρ = 0.548, *p <* 0.05) induced higher soil nutrient contents (ρ = 0.485, *p <* 0.05) and subsequently increased the soil bacterial diversity and richness (ρ = 0.502, *p <* 0.01), which increased the POC content (ρ = 0.525, *p <* 0.01) and indirectly contributed to SOC accumulation. Under the O+M treatment, the soil nutrients (ρ = 0.485, *p <* 0.05) and the microbial C and P limitation (ρ = −464, *p <* 0.01) had significant direct effects on the soil bacterial diversity and richness. On the other hand, the soil microbial C and P limitation (ρ = −0.546, *p <* 0.01) had significant direct effects on the K/r strategy of bacteria. Elevated soil bacterial diversity and richness (ρ = 0.502, *p <* 0.01) and K/r strategy (ρ = 0.428, *p <* 0.01) enhanced POC, indirectly boosting SOC sequestration. In addition, the O+M treatment (ρ = −607, *p <* 0.01) reduced the soil salt and pH, which elevated both POC and MAOC (ρ = 0.398, *p <* 0.05) to further augment the SOC accumulation indirectly (*p* < 0.05).

## 4 Discussion

### 4.1 Response of the SOC pool to organic ameliorants

The application of organic ameliorants significantly enhanced the SOC content (**Figure 2**), consistent with our first hypothesis. This increase occurred through two pathways: i) direct SOC addition from the returned organic materials serving as carbon substrates (Song et al., 2023; Zhang et al., 2024) and ii) provision of abundant carbon and energy substrates for soil microorganisms, which stimulated the secretion of extracellular enzymes to utilize the exogenous organic matter (Nannipieri et al., 2012; Zhu et al., 2022). In addition, the organic amendments improved the saline–alkali environment, thereby fostering more favorable conditions for SOC accumulation. This improvement likely resulted from the enhanced soil structure and permeability, which facilitated the leaching of saline ions (e.g., Na^+^ and Cl^−^), combined with the neutralizing effect of organic acids (Zhang et al., 2020; Li et al., 2024b). The reduction in salinity and pH alleviated the stress on the soil microorganisms, promoted their proliferation, and enhanced the soil enzyme activities (Che et al., 2023). Collectively, these modifications improved the saline–alkali environment, fostering more favorable conditions for SOC accumulation (Li et al., 2023). In support of this interpretation, a lower soil salinity, a reduced pH, an elevated enzyme activity, and their strong correlations with SOC were observed (**Figures 1**, **3**; **Supplementary Figure S1**).

The O+M treatment yielded significantly higher SOC content than the O treatment (**Figure 2**), suggesting greater SOC accumulation. This synergy was attributed to the multi-functionality of the applied microbial consortium (containing Actinomycetes and *B. amyloliquefaciens*), which enhanced the transformation and decomposition of exogenous organic materials, thereby contributing to the increased SOC (O’Callaghan et al., 2022; Bamdad et al., 2022). Specifically, the O+M treatment improved the soil nutrient availability. The enhanced nutrient availability (i.e., AP and AK) alleviated the microbial C and P limitations (**Figures 2**, **3**), thereby accelerating the microbial turnover and the subsequent SOC formation. This differential impact was further reflected in the SOC fractions: the O treatment increased only the POC content, whereas the O+M treatment increased both POC and MAOC (**Figure 2**). This divergence likely stems from O+M enhancing the microbial environment, stimulating the microbial secretion and residue production to provide precursor substances for MAOC formation (Mao et al., 2024; Zhou et al., 2024). Concurrently, the lower soil pH and salt content under O+M strengthened the organo-mineral associations (Chi et al., 2022), enhancing MAOC stabilization. Collectively, these results demonstrate that organic ameliorants combining microbial agents effectively boost a stable SOC sequestration.

### 4.2 Regulatory pathways of fertilization management on SOC through soil biotic and abiotic indexes

This study elucidated the factors influencing SOC and its regulatory pathways under different organic ameliorants. SOC, POC, and MAOC showed significant correlations with the soil nutrients, soil salt, pH, and microbial C and P limitations (**Supplementary Figure S1**), which align with previous studies demonstrating that organic ameliorants facilitate SOC sequestration by improving the soil nutrients and saline–alkali conditions (Li et al., 2023). In this study, organic ameliorants also increased the soil bacterial richness and diversity, particularly of Actinomycetes and Acidobacteriota, which was mainly associated with the improvements in the soil salt, pH, and nutrient status (**Figure 5**; **Supplementary Figure S1**). This is because Actinomycetes, which participate in organic material decomposition and soil carbon cycling, had their growth and reproduction augmented by exogenous organic inputs (Hozzein et al., 2019). Acidobacteriota, as acidophilic bacteria, experience growth inhibition in saline–alkali soil (Li et al., 2021). The reduction in soil pH under organic ameliorants alleviated this stress, thus promoting an increase of Acidobacteriota (Haj-Amor et al., 2022). The concurrent increase of Actinomycetes and Acidobacteriota signifies an increase in K-strategist bacteria. With regard to soil fungi, the microbial agents composed predominantly of bacteria (e.g., *Bacillus* and Actinomycetes) introduced competitive pressure on the indigenous soil fungi for resources. This competition may explain the lower fungal richness observed under the O+M treatment compared with the O treatment. The differential responses of the r- and K-strategist bacteria resulted in a higher bacterial K/r ratio in the O+M than that in the O treatment (**Figure 5**). This indicates that organic ameliorants combining microbial agents shifted the soil bacterial community toward greater dominance by K-strategists.

The soil POC, MAOC, and SOC were strongly correlated with the soil bacterial communities, but weakly correlated with the soil fungal communities, indicating that bacteria are the key microbial drivers of the SOC pool dynamics. The organic amendments indirectly increased SOC by elevating POC and MAOC, which are regulated by biotic and abiotic factors. Specifically, the application of organic ameliorants elevated the soil nutrient levels, promoting the bacterial diversity and richness. These shifts facilitated more rapid microbial metabolic activities, leading to a higher POC content and contributing indirectly to SOC sequestration (Liu et al., 2022). Simultaneously, the O+M treatment with the reduction of the microbial C and P limitations promoted increases in the soil bacterial diversity, richness, and K/r strategy (**Figure 6**). This is supported by the organic ameliorants that combined microbial agents providing abundant carbon and phosphorus nutrients and promoting the transformation of immobilized phosphate, which i) resulted in higher microbial abundance and activity (Li et al., 2024c) and ii) alleviated the soil C limitation while increasing the stoichiometric N demand, shifting the community toward K-strategy (Kamble and Bååth., 2014; Li et al., 2024d). An increased K/r ratio can improve the microbial resource use efficiency and promote the assimilation of plant-derived carbon into the soil, contributing to POC formation (Morrissey et al., 2023). In addition, a negative path coefficient from O+M treatment to soil salt and pH and a negative path coefficient from soil salt and pH to POC and MAOC were recorded (**Figure 6**). This result implied that the O+M treatment promoted POC and MAOC formation by reducing the soil salt content, indirectly contributing to SOC sequestration. There are three potential reasons for this phenomenon: firstly, the O+M treatment alleviates the hindering effect of high salinity on soil particle cementation, favoring POC accumulation (Hassani et al., 2024). Secondly, the lower soil salt content decreases the competition of the base ions (e.g. Na^+^, HCO_3_^−^, and Cl^−^) for the binding sites of soil minerals and organic materials (Chi et al., 2022; Kleber et al., 2021). Thirdly, the dissolution of phosphate precipitation is stimulated by a reduction in the soil pH, which may increase the soil Ca^2+^ and Mg^2+^ ions, thus enhancing the adsorption of soil minerals on organic carbon and promoting MAOC formation (Rowley et al., 2018). Overall, a single application of organic ameliorants increased the SOC sequestration indirectly by increasing POC. Organic ameliorants combined with microbial agents indirectly increased the SOC sequestration through the concurrent increase of both POC and MAOC. This mechanistic distinction explained our finding of the contribution of MAOC to the SOC increment under the O+M treatment being higher than that under the O treatment.

## 5 Conclusion

This study demonstrated that the synergistic combination of organic ameliorants and microbial agents (O+M) proved substantially more effective than an organic amendment alone (O) for enhancing SOC sequestration. While O primarily increased POC, O+M uniquely enhanced both POC and MAOC. This divergence stems from the introduced microbial inoculants (Actinomycetes and *B. amyloliquefaciens*) accelerating the exogenous organic matter transformation. The reduction of the soil salt content and pH created favorable physicochemical conditions for the formation and stabilization of MAOC. Simultaneously, the alleviation of the microbial carbon and phosphorus limitations promoted a shift in the bacterial community toward K-strategists (e.g., Actinomycetes and Acidobacteriota). This K-strategy dominance enhanced the microbial resource use efficiency, facilitating residue transformation into SOC. In summary, while organic ameliorants effectively primarily boost SOC through increasing the POC, their synergistic integration with microbial agents creates a unique microenvironment to increase SOC accumulation through the co-increase of the POC and MAOC fractions. Thus, the combined application of organic ameliorants and microbial agents is established as the superior strategy for increasing SOC in saline–alkali soils.

## Data Availability

The original contributions presented in the study are included in the article/**Supplementary Material**. Further inquiries can be directed to the corresponding authors.
