# Supplementary material for: Mitigating soil salinity–alkalinity and reshaping bacterial community to improve soil organic carbon sequestration in the Hetao Irrigation District: a combined approach of organic ameliorant and microbial agents
Source: Front Plant Sci. 2026 Feb 4;17:1754594. doi: 10.3389/fpls.2026.1754594 (PMC12915042; doi:10.3389/fpls.2026.1754594)
Supplement: Supplementary file 1 [file DataSheet1.docx]

**Supplementary materials**

**Materials and methods**

*Soil microbial community structure analysis*

Briefly, the soil DNA was extracted using the E.Z.N.A.® DNA Kit (OmegaBio-tek, Norcross, GA, U.S.). The extracted genomic DNA was used as the template to amplify the hypervariable ITS region of fungi with primers ITS3(5'-GCATCGATGAAGAACGCAGC-3') and ITS4 (5'-TCCTCCGCTTATTGA TATGC-3'), and amplify the V3-V4 region of 16S rRNA genes with primers 338 forward (5′-ACTCCTACGGGAGGCAGCA-3′) and 806 reverse (5′-GGACTACHVG GGTWTCTAAT-3′). PCR purification was conducted using 2 % agarose gel and AxyPrepDNA gel recovery kit (AXYGEN). Then, PCR products were sequenced on the Illumina platform. The soil DNA was extracted using the E.Z.N.A.® DNA Kit (OmegaBio-tek, Norcross, GA, U.S.). The extracted genomic DNA was used as the template to amplify the hypervariable ITS region of fungi with primers ITS3(5'-GCATCGATGAAGAACGCAGC-3') and ITS4 (5'-TCCTCCGCTTATTGA TATGC-3'), and amplify the V3-V4 region of 16S rRNA genes with primers 338 forward (5′-ACTCCTACGGGAGGCAGCA-3′) and 806 reverse (5′-GGACTACHVG GGTWTCTAAT-3′). PCR purification was conducted using 2% agarose gel and AxyPrepDNA gel recovery kit (AXYGEN). Then, PCR products were sequenced on the Illumina platform.


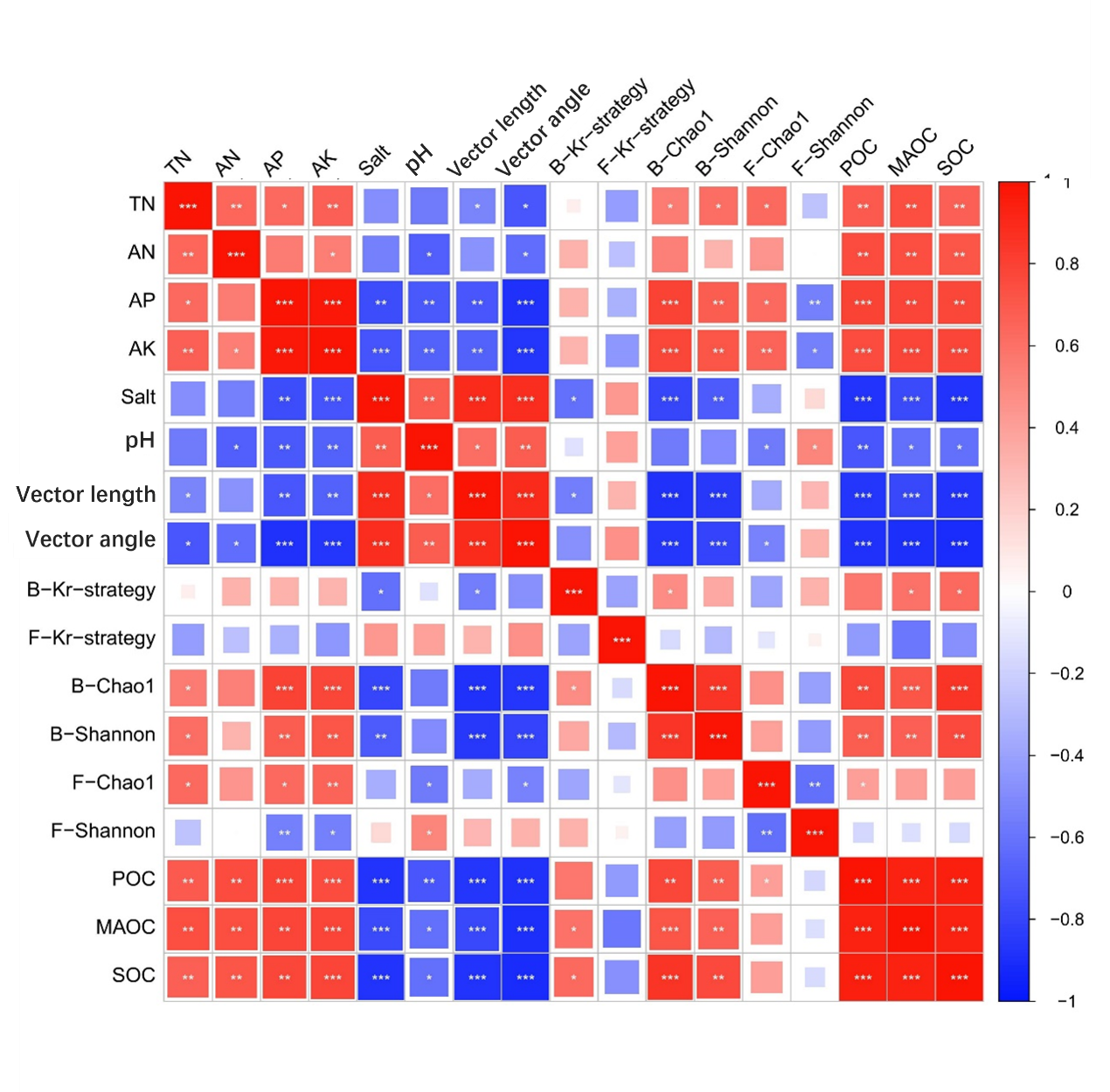


**Figure S1** Correlation between SOC pool and soil nutrients, salt, pH, enzyme activities and microbial communities. TN, total nitrogen; AN, available nitrogen; AP, available phosphorous; AK, available potassium, Salt, salt content; POC, particulate organic carbon; MAOC, mineral-associated organic carbon; SOC, soil organic carbon, B, bacterial; F, fungal.
